# Microstructure and Performance Evolution of Post-Plastic Deformed Austenitic Stainless Steel Fabricated by Selective Laser Melting

**DOI:** 10.3390/mi16101104

**Published:** 2025-09-28

**Authors:** Huimin Tao, Zi Li, Linlin Ma, Yafang Cai, Haiteng Xiu, Mingming Ding, Zeqi Tong

**Affiliations:** 1 Nanxun Innovation Institute, Zhejiang University of Water Resources and Electric Power, Hangzhou 310018, China; p24010858008@cjlu.edu.cn (Z.L.);; 2 College of Mechanical and Electrical Engineering, China Jiliang University, Hangzhou 310018, China; 3 Key Laboratory of Special Equipment Safety Testing Technology of Zhejiang Province, Zhejiang Academy of Special Equipment Science, Hangzhou 310020, China

**Keywords:** additive manufacturing, stainless steel, stretch deformation, microstructure, mechanical properties, corrosion properties

## Abstract

With the rapid development of additive manufacturing technology, selective laser melting (SLM) of austenitic stainless steel has been widely used. SLM stainless steel will inevitably deform during service, so it is necessary to study the microstructure and macro properties of post-plastic deformed SLM stainless steel. In this paper, the changes in the microstructure, mechanical properties, and corrosion resistance of SLM304 stainless steel after stretch deformation were studied, and the evolution rules were revealed. The results show that, with an increasing plastic deformation amount, SLM304 stainless steel exhibits grain fragmentation, disordered orientation, and subgrain formation, along with changes in the shape and size of the cellular structure. Additionally, the α’ martensite content inside SLM304 stainless steel rises significantly, while the thickness of the surface passivation film slightly decreases. The analysis shows that the combined effect of the complex microstructure makes the nanohardness of SLM304 stainless steel increase with the increase in the stretch deformation amount while its corrosion resistance deteriorates. Therefore, moderate post-plastic deformation can enable SLM stainless steel to balance excellent mechanical and corrosion properties. This study can not only provide a theoretical reference for the performance optimization of additive manufacturing steel but also provide value for the engineering application of additive manufacturing technology.

## 1. Introduction

As a metal material with excellent corrosion resistance, mechanical properties, and processability, stainless steel is widely used in aerospace, medical devices, energy equipment, and other high-end manufacturing fields. Traditional stainless steel processing relies on casting, forging, cutting, and other material reduction processes, which have the limitations of low material utilization, difficulty in forming complex structures, long production cycle, and so on. Additive manufacturing technologies, especially selective laser melting (SLM), electron beam melting, and other powder bed melting technologies, directly form near net shape parts by stacking materials layer by layer, breaking through the design limitations of traditional processes on complex structures, significantly improving material utilization and production flexibility, and providing a new solution for the high-performance and lightweight manufacturing of stainless steel components [1,2].

However, during SLM, stainless steel powder undergoes rapid melting and solidification cycles under the action of high-energy lasers or electron beams, resulting in extremely high temperature gradients and non-uniform thermal stress in local areas [3]. During this process, rapid melting and solidification and a complex thermal history can easily lead to micro defects (such as residual stress, porosity, and cracks) and non-equilibrium structures (such as metastable austenite, high-density dislocation, cellular structure, and weld pool boundary) during and after forming [4]. In addition, the deformation of stainless steel produced by the additive is inevitable in the process of workpiece forming and service. This deformation problem not only reduces the dimensional accuracy and surface quality of parts but also leads to the degradation or even failure of the service performance of workpieces, which has become one of the core bottlenecks restricting the industrial application of additive manufacturing stainless steel technology.

In recent years, scholars at home and abroad have carried out considerable research on the deformation mechanism of stainless steel with additive materials. On the one hand, through the establishment of a thermal–mechanical coupling numerical model (such as finite element method, discrete element method) to simulate the dynamic evolution of the molten pool, stress field distribution, and deformation law, the influence mechanism of process parameters (laser power, scanning speed, layer thickness, etc.), scanning strategy (such as zone scanning, rotating scanning), and material characteristics (powder particle size, thermophysical parameters) on deformation behavior is revealed [5]. On the other hand, by optimizing the process path (such as preheating the substrate, variable power scanning), designing the supporting structure, and implementing the subsequent heat treatment (such as stress relief annealing), the deformation suppression method is explored [6]. Although some progress has been made, the existing research focuses on the mechanism, model prediction, and control strategy of the influence of printing process parameters on the deformation of the workpiece, while little research has been performed on the deformation situation and mechanism of additive manufacturing stainless steel after printing. It is difficult to meet the manufacturing and application requirements of stainless steel workpieces with high requirements and complex structures in the actual production of additive manufacturing stainless steel. However, the special and complex microstructure of additive manufacturing stainless steel makes the material show a different structural evolution and performance response from the traditional processing material in the subsequent plastic deformation. Compared with forged stainless steel, SLM stainless steel has excellent tensile ductility and a doubled yield strength due to element segregation and unique honeycomb substructure. The author believes that during the deformation process of SLM stainless steel, in addition to obvious deformation twinning, deformation faults and dislocation cell refinement are significant features of the entire tensile deformation. The synergy of these mechanisms dominates the deformation behavior at low strain levels, while deformation faults and twinning play a crucial role at medium to high strain levels. These deformation mechanisms together result in a stable strain hardening rate during the tensile process, thereby endowing SLM stainless steel with excellent tensile ductility and a high yield strength [7,8]. There is little research in this area, and further research is needed.

Under this background, this study took 304 stainless steel, commonly used in production and life as the research object, and studied the evolution of the microstructure and macro properties of SLM304 stainless steel under different deformation amounts after tensile deformation. At the same time, the effect of the microstructure on the mechanical properties and corrosion resistance of SLM stainless steel and the correlation between the microstructure and properties were analyzed. The evolution mechanism of the microstructure and properties of SLM304 stainless steel with different deformation amounts was revealed. This study not only provides a theoretical reference for the performance optimization and wide application of SLM stainless steel but also provides value for the application of additive manufacturing technology.

## 2. Materials and Methods

### 2.1. Material Preparation and Stretch Deformation Process

**Figure 1:**
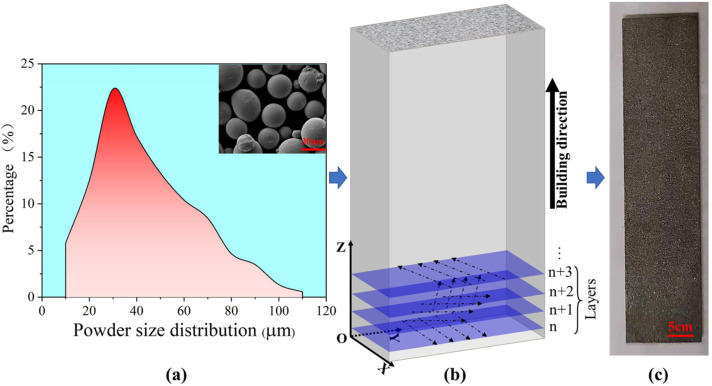
Preparation of 304 stainless steel fabricated by selective laser melting. (**a**): Stainless steel powder; (**b**): stainless steel printer schematic diagram; and (**c**): printed sample drawing.

**Figure 2:**
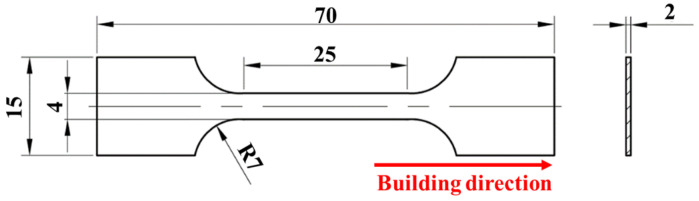
Dimensions of stretched samples (mm).

The preparation process of SLM304 stainless steel used in this study is shown in Figure 1. Figure 1a shows the situation of metal powder, which is used to prepare a material sheet by the EOS M290 (Electro Optical Systems GmbH, Munich, Germany) machine, and the equipment is equipped with a 400 W Yb: YAG fiber laser. The key printing process parameters are as follows: 200 W laser power, 950 mm/s scanning speed, 40 μm powder layer thickness between layers, 0.07 mm scanning spacing, and 67° scanning direction change. Figure 1b shows the printer schematic, and the sheet metal in Figure 1c is printed. After printing is completed, in order to eliminate residual stress inside the 304 stainless steel, the material is heated to 450 °C in a heating furnace for 3 h and finally cooled to room temperature in the heating furnace. The measured chemical composition of SLM304 steel is shown in Table 1. The tensile mechanical properties of printed SLM304 stainless steel were shown in Table 2. The samples were cut into stretch samples by wire electrical discharge machining, and the dimensions are shown in Figure 2. The size ratio relationship follows the requirements of the standard test method for tensile testing of metal materials (ASTM E8/E8M [9]).

**Figure 3:**
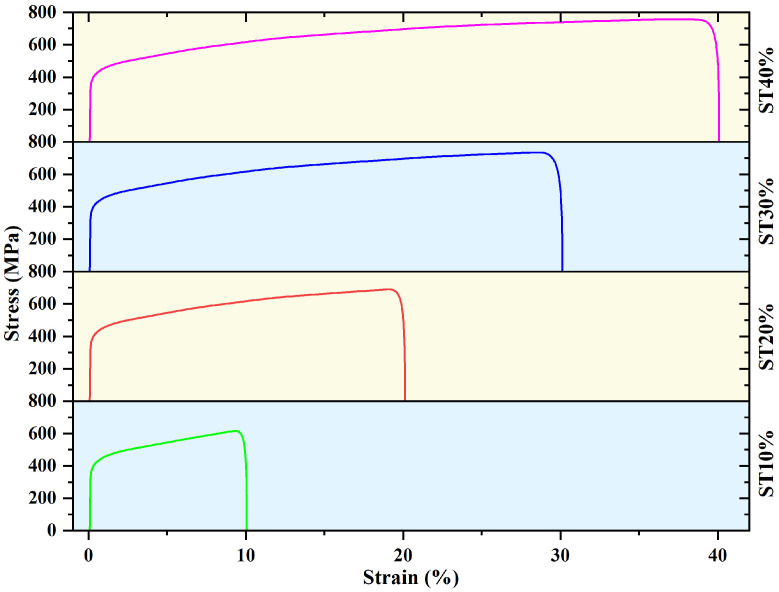
Stress–strain curve of SLM304 stainless steel with different stretch deformation amounts.

After the thin plate material is prepared, the material is processed into a slow strain rate tensile specimen by wire cutting equipment, and the dimension is shown in Figure 2. The tensile test of 304 stainless steel was carried out by an Instron 8801 universal testing machine (Instron Corporation, Norwood, MA, USA) equipped with an extensometer. The research shows that the elongation of SLM304 stainless steel can exceed 50% [10]. The deformation set in the tensile experiment in this study is 10%, 20%, 30%, and 40%, respectively, and the tensile direction is along the direction of printing. The deformation velocity of all samples during tension is 5 × 10^−5^ s^−1^. In this paper, the names of undeformed and different deformation samples are ST0%, ST10%, ST20%, ST30%, and ST40%, respectively. The stretch test under each condition was repeated three times to ensure the stability of the test results. Figure 3 shows the stress–strain curves of SLM304 stainless steel after tensile deformation under different conditions. After the stretch test, the microstructure and macro property evolution of SLM stainless steel with different deformation amounts in the middle of the sample were observed.

### 2.2. Microstructure Characterization and Macroscopic Performance Testing

An optical microscope (OM) was used to observe the metallographic appearance of different samples. A field emission scanning electron microscope (FESEM) was used to explore the microscopic cellular structure of the samples. At the same time, electron backscatter diffraction (EBSD) was used to reveal the grain structure, orientation, and distribution of the samples. In addition, X-ray photoelectron spectroscopy (XPS) and a ferrite equivalent instrument (FQI) were used to analyze and study the surface film and phase structure. The FQI quantifies the total content of ferromagnetic phases in a material by measuring its magnetic permeability. The higher the content of ferromagnetic phase (martensite) in 304 stainless steel, the stronger the overall magnetic properties of the material. The acceleration voltage of the EBSD test is 20 kV, the scanning speed is 635 Hz, and the scanning step is 1 μm. The electron energy of the XPS test electron source is 2000 eV, the energy half-width is 0.4 eV, the minimum beam spot diameter is about 1 mm, and the maximum output current is about 65 μA.

The nanoindentation test is used to analyze the mechanical properties of materials on the micro and nanoscale, and the hardness is measured by a high-precision indentation method [11]. Before the nanoindentation test, all samples were electropolished in a 90% CH_3_COOH and 10% HClO_4_ solution to obtain smooth surfaces. The Agilent G200 nanoindentation system (KLA Instruments, Milpitas, CA, USA) equipped with a Berkovich diamond tip was used for a nanoindentation test at room temperature. The Agilent G200 nanoindentation instrument is force controlled, so the test is controlled by the amount of loading. The maximum load of each indentation test is 1.0 mN, and the holding time is 5 s. At least five locations were selected on each specimen for testing, and the spacing between each indentation is 1 mm, which is large enough to avoid the interaction between the indentation, and the load displacement curve is observed. Finally, the average nanohardness value is calculated, and the formula used is H = P_max_/A_c_ (P_max_: maximum compression load, A_c_: actual contact projection area between the indenter and the material) to calculate the nanohardness result [12].

Electrochemical corrosion tests were carried out on the stretch samples. The open circuit potential (OCP) test and dynamic polarization (DP) test of materials with different deformation treatments were tested by an electrochemical workstation to study the corrosion resistance of different samples. During electrochemical measurement, each sample was embedded in the plastic pipe through epoxy resin, and the rear end of the sample was welded with copper wire. The working area is a circular area with a diameter of 10 mm, which is exposed to the solution. All samples were successively polished with sandpaper from 400 to 2000 mesh and then polished with 0.5 μm and 0.1 μm diamond polishing agents to obtain a smooth surface. Clean the sample surface with ultrasonics before the experiment to ensure a clean performance. The experiment was carried out using an Ivium SXRE (Ivium Technologies B.V., Eindhoven, The Netherlands) electrochemical workstation with the software of IviumSoft 4.1216. The workstation with a stainless steel sample as the working electrode, a platinum plate as the counter electrode, and 232 saturated calomel electrodes (SCE) as the reference electrode. All electrochemical measurements were carried out in a constant temperature water bath at 25 ± 1 °C. The OCP test lasted for about 2 h. Before the DP experiment, put the sample in the solution for about 10 min to make the experiment more stable. The DP experiment was carried out in 3.5% (weight percentage) sodium chloride electrolyte solution. The DP curve was obtained by scanning from (−1.0) V_SCE_ to (+1.0) V_SCE_ at the rate of (+0.1) mV/s. Each test shall be repeated at least five times to ensure its reliability. After testing, the experimental result curve and the distribution of pitting corrosion were observed, and the effect of different deformations on the corrosion resistance of stainless steel was analyzed.

## 3. Results

### 3.1. Microstructural Characterization

**Figure 4:**
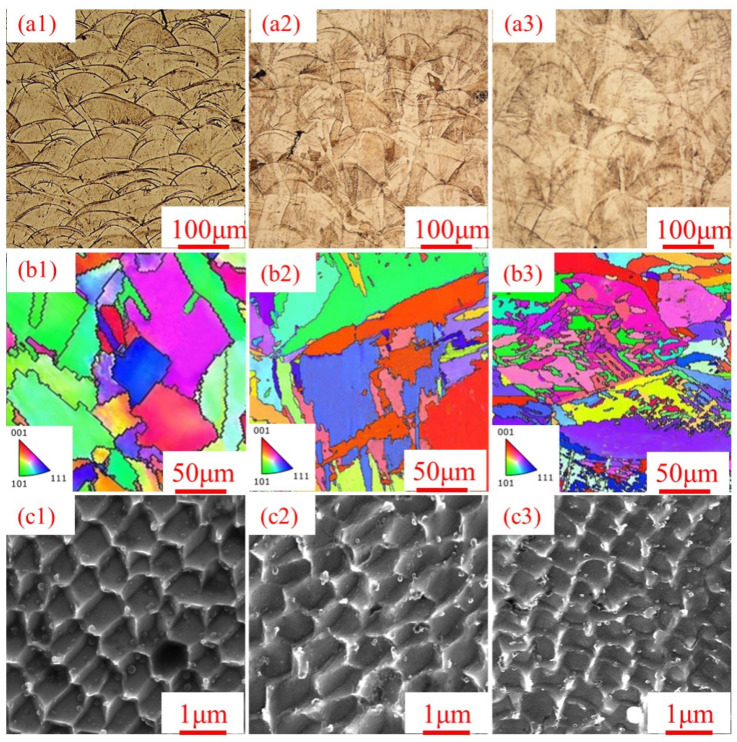
Metallography of SLM304 stainless steel with different stretch deformation amounts. (**a1**–**a3**): Metallography; (**b1**–**b3**): inverse pole figure of EBSD; and (**c1**–**c3**): field emission scanning electron microscope image. ((**a1**–**c1**): 0%; (**a2**–**c2**): 20%; and (**a3**–**c3**): 40%).

Figure 4 shows the microstructure evolution of SLM304 stainless steel with different stretch deformation amounts. Figure 4(a1–a3) presents the metallography of SLM304 stainless steel with different deformation amounts. Observe the undeformed SLM304 stainless steel in Figure 4(a1). The SLM stainless steel sample is composed of very regular fan-shaped molten pools along the same direction, with different sizes of molten pools. After stretch deformation with an amount of 20% (Figure 4(a2)), the weld pool morphology in SLM stainless steel can still be observed, but the corrosion degree of the weld pool boundary is reduced. Some weld pool boundaries become blurred, and the shape of the weld pool boundary is damaged. At the same time, the morphology of irregular grains can be observed. When the stretch deformation amount is large (40%) (Figure 4(a3)), the molten pool of SLM stainless steel is deformed, the boundary is obviously damaged and becomes very fuzzy and chaotic. At the same time, there are many fine subgrain clusters on the surface. It can be seen that the molten pool and grain structure of SLM304 stainless steel will change significantly when stretch deformation occurs, and the degree of structural damage will increase with the increase in deformation.

Figure 4(b1–b3) shows the reverse pole diagram of the EBSD test of SLM 304 stainless steel with different deformation amounts. It can be seen that the microstructure of SLM304 stainless steel changes significantly with the stretch deformation. Generally, the grain boundary of traditional forged stainless steel is a regular straight line [13]. In contrast, the grain boundary of the initially printed undeformed SLM304 stainless steel is very irregular, resulting in a very irregular morphology of the grains (Figure 4(b1)). At the same time, the orientation of each grain in the material is relatively uniform, but there are many grain orientations as a whole. As the deformation amount increases to 20% (Figure 4(b2)), it can be seen that the sample has irregular polygonal grains similar to the undeformed sample, and a few fine subgrain structures appear inside the grains, and the grain orientation becomes chaotic. When the deformation reaches 40% (Figure 4(b3)), it can be seen that the grains are obviously destroyed, and a large number of subgrain structures are generated in the grains. Moreover, the grain orientation obviously becomes very disordered, which indicates that large deformation makes the material structure disordered, which is consistent with the metallographic results.

Figure 4(c1−c3) shows the honeycomb polygonal cellular structure of SLM stainless steel observed under FESEM. The unique structure is formed due to the non-equilibrium metallurgical behavior during the rapid solidification process of SLM [14]. It was found that stretch deformation significantly changed the microcellular structure of SLM304 stainless steel. The section of the cellular structure of the undeformed SLM304 stainless steel is a regular polygon (Figure 4(c1)), and the interface of the cellular structure is very clear under the electron microscope. As the stretch deformation increases to 20% (Figure 4(c2)), it can be seen that the shape of the cellular structure of the sample changes little, but the size of a single cellular structure decreases slightly, and the boundary of the cellular structure becomes blurred in the same case. When the deformation reaches 40% (Figure 4(c3)), the shape of the cellular structure of the sample becomes chaotic, the boundary of the cellular structure becomes very fuzzy, and the size of a single cellular structure is significantly reduced. It can be seen that the deformation has a significant effect on the micron structure of SLM304 stainless steel.

**Figure 5:**
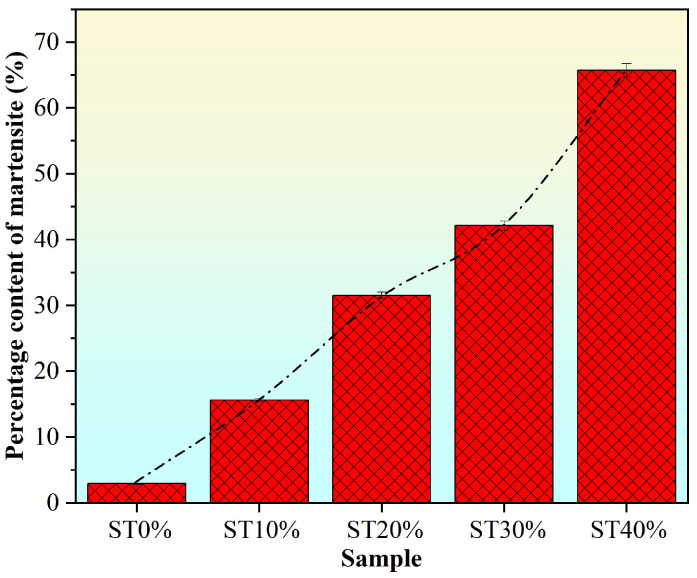
Martensitic content of SLM304 stainless steel with different stretch deformation amounts.

In addition, to analyze the phase change of 304 stainless steel during deformation, the content of phase-change martensite was measured using a ferrite equivalent meter, and the results are shown in Figure 5. The results show that the martensite content of ST0%, ST10%, ST20%, ST30%, and ST40% are 2.9%, 15.6%, 31.5%, 42.1%, and 65.7%, respectively. These results indicate that the initial printed 304 stainless steel contains a small amount of martensite, and the content of martensite gradually increases with the increase in deformation. The martensitic transformation degree of SLM304 stainless steel is small under small deformation. With the increase in deformation, the martensite content increases gradually. Moreover, compared with the small deformation, the martensite content of SLM304 stainless steel increases faster when the deformation is large. When the large deformation amount is 40%, the content of martensite is obviously higher than that of austenite. The content of austenite and martensite in stainless steel will have an important influence on its properties.

### 3.2. Surface Passive Film Characteristics

**Figure 6:**
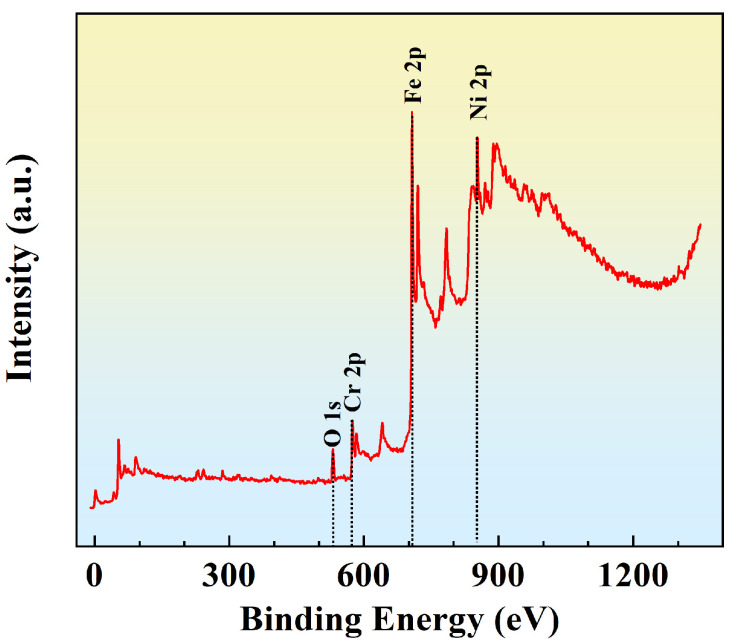
XPS study of passivation film formed on the surface of undeformed SLM304 stainless steel in NaCl medium.

In order to analyze the corrosion behavior of stainless steel, XPS was used to analyze the composition of the passive film on the surface of printed SLM304 stainless steel after anodic voltage polarization in a NaCl medium. The results are presented in Figure 6. The peaks of Fe, Cr, Ni, and O were found in XPS. The results show that the passive film on the surface of SLM304 stainless steel is composed of components related to these elements. This finding is consistent with the traditional forged 304 stainless steel [15].

**Figure 7:**
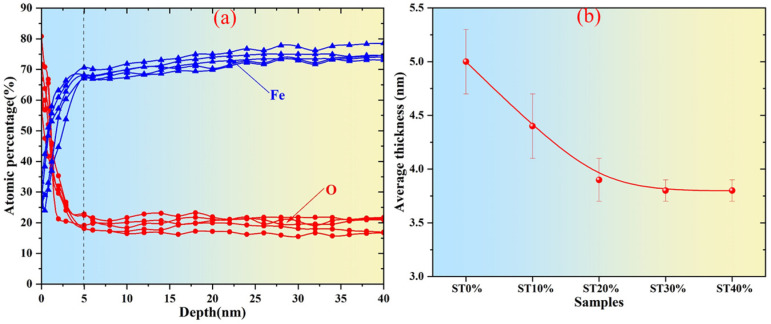
Test result graph of the thickness of the passivation film on SLM304 stainless steel in NaCl medium. (**a**): Distribution diagram of Fe and O elements of undeformed SLM304 stainless; (**b**): average thickness test results of SLM304 stainless with different stretch deformation amounts.

In order to further analyze the thickness of the passive film on the surface of SLM304 stainless steel, the ion etching technology of XPS was used. Generally, the passivation film on the surface of stainless steel is mainly oxide, so the content of oxygen in the film is significantly higher than that of the metal substrate. However, the content of Fe in the matrix must be significantly higher than that in the surface film [16]. Based on this theory, the thickness of the passive film on the surface of stainless steel can be measured by etching from the surface of stainless steel to the inside with high-energy ions in XPS and observing the change in element content. The test results of passive film thickness on the surface of SLM304 stainless steel initially printed are shown in Figure 7a. It can be seen from the figure that with the increase in ion etching depth, the content of the O element gradually decreases, and at a depth of about 5 nm, the content of the O element basically remains stable. For the content of the Fe element, with the increase in the ion etching depth, the content also shows a significant turning point at about 5 nm and maintains a higher content value at a deeper position. Therefore, it can be seen from the changes in the O and Fe content in the results that the average thickness of the printed passive film on the surface of SLM304 stainless steel is about 5 nm. Using the same method, the thickness of the passive film on the surface of SLM304 stainless steel with different deformations is tested and counted, and the results are shown in Figure 7b. The results show that the thickness of the passive film on the surface of SLM304 stainless steel gradually decreases with the increase in stretch deformation. When the deformation is less than 20%, the film thickness decreases rapidly; meanwhile, when the deformation is more than 20%, the film thickness decreases slowly. When the deformation is 30%, the average thickness value of the passive film is only about 3.7 nm. When the deformation continues to increase, the thickness of the passive film remains basically unchanged. It can be seen that the stretch deformation has a significant effect on the thickness of the passive film on the surface of SLM304 stainless steel. The change in passive film on the surface of SLM304 stainless steel will further affect its corrosion resistance.

### 3.3. Mechanical Nanoindentation Testing

**Figure 8:**
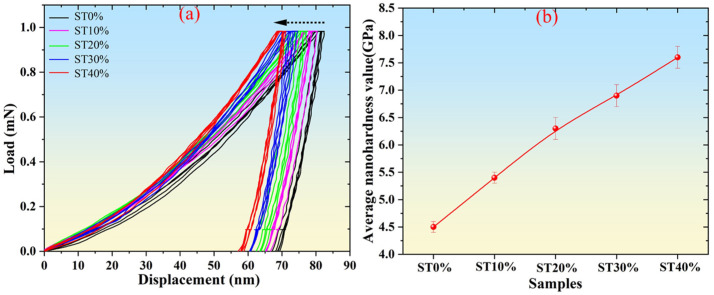
Nanoindentation test results of SLM304 stainless steel with different stretch deformation amounts. (**a**): Nanoindentation load–displacement curves; (**b**): nanohardness variation graph.

The nanoindentation test is an advanced technology for measuring the mechanical properties of materials at the nano scale. It can obtain the parameters such as nanohardness by pressing the micro indentation on the surface of materials [17]. In order to analyze the micro-mechanical properties of SLM304 stainless steel under different stretch deformations, a nanoindentation test was carried out on the samples. Figure 8 shows the nanoindentation test results of SLM304 stainless steel with different deformation amounts. It can be seen from the load displacement curve in Figure 8a that when the load applied to different samples increases to the maximum value of 1 mN and remains at the maximum value, the maximum depth of indentation of different samples is significantly different. The observation shows that with the increase in the deformation amount, the load displacement curve obviously moves to the left, and the maximum indentation depth obviously decreases, indicating that the nanohardness of the sample gradually increases. Figure 8b is a statistical diagram of the average nanohardness values of SLM304 stainless steel with different deformation amounts. The results show that the average nanohardness value of the original printed SLM304 stainless steel is about 4.5GPa, and the average nanohardness value of stainless steel increases gradually with the increase in deformation. When the deformation reaches 40%, the average nanohardness reaches about 7.5 GPa. The results of the nanohardness calculation are consistent with the results of the load–displacement curve.

### 3.4. Corrosion Resistance Testing

In addition to mechanical properties, the corrosion performance of metals in use is also very important. In order to reveal the changes in corrosion properties of SLM304 stainless steel after stretch deformation, electrochemical corrosion tests were carried out on samples with different deformation amounts. Electrochemical tests include the OCP test and DP test. In the OCP test, the higher the open circuit voltage of the sample, the better the corrosion resistance. In the DP test, the corrosion potential (E_cos_) and corrosion current density (I_cos_) directly reflect the spontaneous corrosion tendency of metals in a corrosive environment. The greater the negative value of the corrosion potential, the greater the corrosion current, indicating that the corrosion tendency of the metal is stronger and vice versa [18]. The pitting corrosion potential (E_pit_) and pitting corrosion current density (I_pit_) reflect the ability of the metal to resist pitting corrosion. The greater the positive value of E_pit_, the smaller the current of I_pit_, indicating that the pitting corrosion resistance of the metal is stronger and vice versa [19].

**Figure 9:**
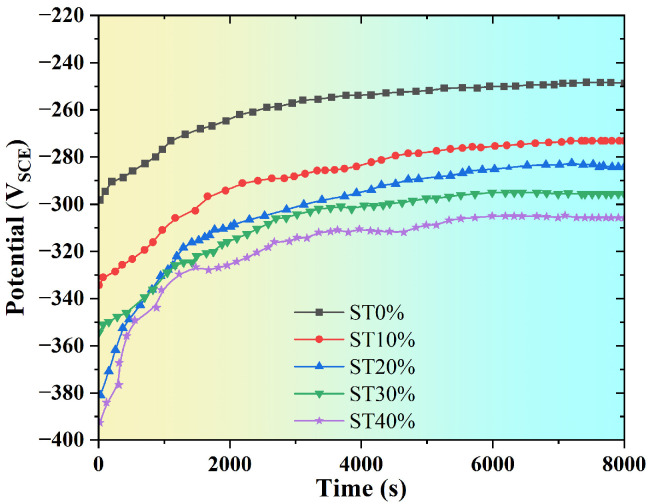
Test results of the electrochemical open circuit potential of SLM304 stainless steel with different stretch deformation amounts.

Figure 9 shows the OCP test results of SLM304 stainless steel with different stretch deformation amounts. It can be seen that all tested samples show the same rule with the change in OCP in the corrosion solution. With the increase in time in the corrosion solution, the OCP increases linearly at first and then remains at a stable voltage for about 3000 s. Compared with the original printed SLM304 stainless steel OCP, the stable OCP measured by the sample after stretch deformation is significantly reduced. It indicates that stretch deformation reduces the corrosion resistance of SLM304 stainless steel. In addition, it was observed that the OCP of the sample gradually decreased with the increase in the stretch deformation amount. Therefore, the results show that the corrosion resistance of SLM304 stainless steel decreases with the increasing deformation amount.

**Figure 10:**
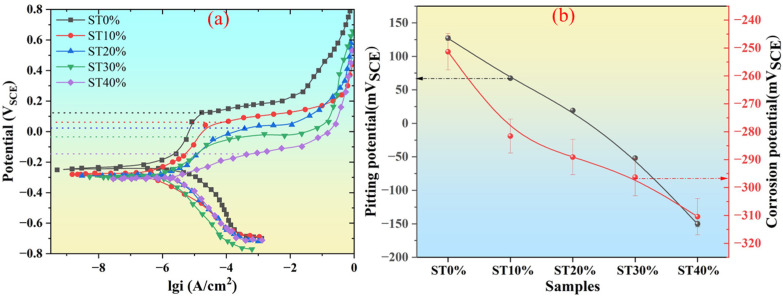
Test results of electrochemical dynamic polarization of SLM304 steel with different stretch deformation amounts. (**a**): Dynamic polarization curve graph; (**b**): potential change graph.

**Figure 11:**
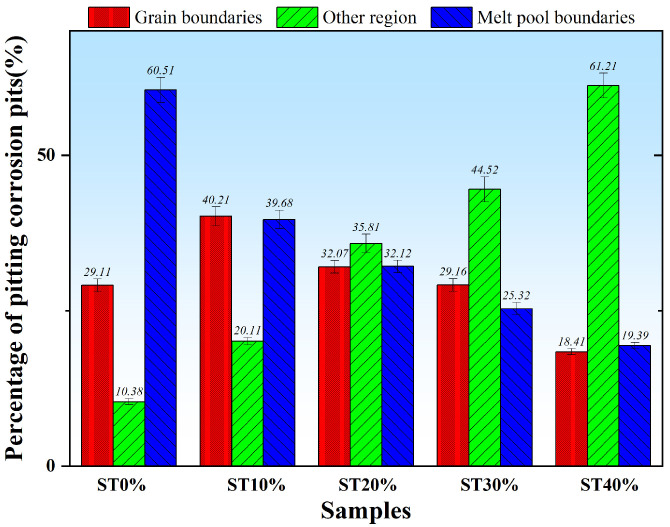
Distribution of corrosion pits after pitting corrosion of SLM304 stainless steel with different stretch deformation amounts.

Figure 10 shows the electrochemical DP test results of SLM304 stainless steel under different stretch deformation conditions. The results show that the shape of the DP curve of SLM304 stainless steel before and after stretch deformation is similar, showing an obvious passivation zone. But in each sample, the key parameters of the DP curve change greatly. The changes in the corrosion voltage and pitting voltage of SLM304 samples with different stretch deformation amounts are shown in Figure 10b. The key parameters of the DP curve are shown in Table 3. The results show that after stretch deformation, the E_pit_ value of the SLM304 stainless steel sample is significantly reduced, indicating that the stretch deformation worsens the pitting corrosion resistance. With the increase in the deformation amount, it was observed that the E_pit_ value gradually decreased, and the I_pit_ value gradually increased, indicating that the pitting corrosion resistance of SLM304 stainless steel became worse. In addition, with the increase in the deformation amount, the passivation area in the polarization curve is significantly reduced, and the passivation area of the ST40% sample with a large deformation is significantly smaller. It indicates that the passivation ability of SLM304 stainless steel decreases with the increase in the deformation amount. The observation of corrosion parameters showed that after stretch deformation, the E_cos_ value of SLM304 stainless steel decreased significantly, indicating that the corrosion resistance of stainless steel became worse after deformation. With the increase in deformation, the E_cos_ value gradually decreases, and the I_cos_ value gradually increases, indicating that the corrosion resistance of 304 stainless steel gradually decreases. This result is consistent with the above OCP test results. The distribution of corrosion pits after the pitting corrosion of SLM304 stainless steel with different stretch deformation amounts is shown in Figure 11. The results show that the pitting corrosion of SLM304 stainless steel mainly occurs at the boundary of the molten pool and the grain boundary when it is not deformed. With the increase in the deformation amount, the pitting corrosion at the boundary of the molten pool and grain boundary decreases significantly. The statistical results show that the weld pool boundary and grain boundary in SLM stainless steel have an important influence on its pitting corrosion performance. Therefore, the stretch deformation reduces the corrosion resistance of SLM304 stainless steel, and with the increase in deformation, the corrosion resistance gradually decreases.

## 4. Discussion

According to the above research results, the microstructure and properties of SLM304 stainless steel undergo significant changes under tensile deformation. With the increase in the deformation amount, the grains become fragmented, and numerous subgrains gradually form within them, accompanied by increasingly disordered grain orientations [Figure 4(a1–a3,b1–b3)]. At a more microscopic level, the unique micron-level cellular structure of SLM stainless steel changes its shape and size with the increase in the deformation amount, and the interfaces become damaged and blurred due to deformation [Figure 4(c1–c3)]. In addition, deformation causes the transformation from austenite to martensite in stainless steel. With the increase in deformation, the martensite content increases significantly (Figure 5), and the transformation degree is positively related to the deformation. Moreover, the deformation of SLM304 stainless steel also affects the characteristics of the passive film on its surface. With the increase in deformation, the thickness of the passive film on the stainless steel surface decreases slightly (Figure 7). The change in the microstructure of SLM304 stainless steel leads to the change in its macro properties. With the increase in deformation, the nanohardness of SLM304 stainless steel gradually increased (Figure 8). However, the corrosion resistance of deformed SLM304 stainless steel deteriorated, and with the increase in deformation, the corrosion resistance gradually deteriorated (Figure 9 and Figure 10). In the following, the changes in the microstructure and properties of deformed SLM304 stainless steel will be further analyzed.

During the forming process of 304 stainless steel by SLM, metastable austenite, high dislocation density, and residual stress are formed in the stainless steel by rapid solidification. There are fine equiaxed crystals at the boundary of the molten pool inside the SLM stainless steel. The inner part of the molten pool is columnar crystal, and the cross-section is a cellular structure [Figure 4(c1–c3)]. In addition, the special forming method of SLM leads to elemental segregation in stainless steel, with Cr/Mo and other elements being enriched at the boundary of the cellular substructure, thereby forming nanoscale composition fluctuations [20,21]. When stretch plastic deformation occurs, the dislocations in SLM stainless steel will multiply, rearrange, and form dislocation cells. At higher deformation levels, the grain boundary and cellular interface will hinder the dislocation movement, and the deformation force will lead to the deformation of the interface that hinders the dislocation movement. Therefore, with the increase in deformation, the shape of the grain and the cellular structure of SLM304 stainless steel change, and the structure becomes chaotic. Large deformations will also cause the grain boundary migration of stainless steel, and local temperature rise during deformation will lead to transformations from a small-angle grain boundary to a large-angle grain boundary [22]. And when the local strain energy is high, equiaxed subgrains are formed through continuous dynamic recrystallization, as shown in Figure 4. A large number of subgrain structures are generated in the grains during large deformation. At the same time, plastic deformation causes the dislocations to slip in the cell, and the cell wall acts as an obstacle to hinder dislocation movement, resulting in dislocation accumulation at the cell wall [23]. The local stress concentration at the cell wall of the cellular structure will cause the initial dislocation reorganization. When the deformation is large, the accumulation of high-density dislocations leads to the gradual blurring or partial disintegration of the cell wall, the discontinuous cellular boundary, and even the complete disappearance of the cellular structure, which evolves into the dislocation cellular structure, that is, the cellular substructure. In addition, during stretch deformation, when the stress reaches the critical stress threshold and the energy provided by the applied stress exceeds the chemical driving force of stainless steel, austenite will be reorganized into α’ martensite through the Shockley incomplete dislocation slip [24]. As shown in Figure 5, with the increase in stretch deformation, a large number of phase transformation martensites are generated in SLM304 stainless steel. In addition, plastic deformation will also affect the formation of the passive film on the surface of stainless steel. Figure 6 shows that the passive film of SLM304 stainless steel is mainly composed of Cr and Fe oxides, with a thickness of about 5nm. The special forming process of SLM stainless steel will introduce a higher defect density (such as oxygen vacancies and microcracks), making the integrity and thickness of the passive film on its surface worse than that of stainless steel formed by traditional methods. During stretch deformation, the dislocation movement inside the stainless steel will form sliding steps on the surface, resulting in local rupture of the surface film [25]. With the increase in deformation, the more serious the rupture phenomenon is, the smaller the average thickness of the measured film is, as shown in Figure 7b. It can be seen that stretch deformation has a significant effect on the microstructure of SLM304 stainless steel, and the change in microstructure will affect its macro properties.

The complex microstructure of the deformed SLM304 stainless steel has an important impact on its mechanical properties. The increase in the nanohardness of SLM304 stainless steel after stretch deformation is mainly related to dislocation strengthening, martensitic transformation, grain refinement, and residual stress evolution. SLM forming technology has led to a high initial dislocation density of stainless steel [26]. Stretch plastic deformation will further lead to a large number of dislocation proliferation and entanglement, forming dislocation cells or subgrain boundaries, hindering subsequent dislocation movement, and finally leading to the strain hardening of stainless steel. And with the increase in the deformation amount, this phenomenon becomes more prominent. At the same time, α’ martensitic transformation is induced during deformation, and the hardness of the α’ phase is significantly higher than that of the γ austenite phase. The martensite structure is a body-centered cubic structure, and the bulk density is lower than that of face-centered cubic austenite. The lattice distortion stress field caused by carbon atoms and substituted atoms in martensite is strong, and the resistance to the dislocation movement is large [27]. The increase in martensite content makes the deformation in the process of nanohardness measurement more difficult, which makes the hardness value increase. With the increase in the deformation amount, the content of the martensite phase with high hardness increases, which also directly improves the overall hardness of the material. In addition, the fine cellular structure formed by the rapid solidification of SLM makes it difficult for the stainless steel to undergo dynamic recrystallization during room temperature deformation, but it is broken into smaller subcrystals (Figure 4). Due to the results of the Hall–Petch effect, the nanohardness of stainless steel increased with the decrease in grain size. The combined effect of the above factors leads to an increase in the nanohardness of SLM304 stainless steel, which increases positively with the increase in the deformation amount.

The microstructure change in deformed SLM304 stainless steel not only affects its mechanical properties but also changes its corrosion properties. Stainless steel formed by SLM technology is prone to porosity or incomplete fusion defects, and further tensile deformation can promote defect propagation into microcracks. However, it is easy to form occluded cells at the crack tip, which accelerates local corrosion [28]. Tensile stress may also cause microcracks to form in the film, which propagate along grain boundaries or defects. In addition, stainless steel formed by SLM technology contains high-density dislocations, which proliferate, move, and accumulate during tensile deformation. The sliding steps and cracks formed by dislocation movement will expose the fresh metal substrate to the corrosive solution, forming local anodes and becoming local electrochemical active sites, which then accelerate the occurrence of pitting corrosion [29]. At the same time, the high-density dislocations formed will become channels for the rapid diffusion of corrosive ions such as Cl^−^, accelerating corrosion. In addition, SLM304 stainless steel produces a large amount of α’ martensite during deformation. The newly formed martensitic phase differs from the surrounding austenitic matrix in terms of the composition, structure, and stress state, resulting in a potential difference between them, causing micro galvanic corrosion and accelerating the dissolution of the martensitic region [30]. In addition, the diffusion rate of the Cr element in the martensitic phase region is low, the regeneration ability of local Cr_2_O_3_ is poor, and the quality of the passive film formed is poor. As shown in the passive film test results in Figure 7, with the increase in deformation, the martensite content in SLM304 increases significantly, while the passive film becomes thinner and thinner. The two-phase interface becomes the preferred corrosion path, which results in the poor continuity and compactness of the passive film on the surface of stainless steel. In addition, stretch deformation will cause a large amount of stress concentration inside the stainless steel, and the repair rate of the passive film in the local stress concentration area will decrease, and Cl^−^ and other corrosive ions in the corrosion solution are more likely to penetrate [31]. The atoms at the boundaries of the molten pool and grain boundary in SLM stainless steel are irregularly arranged and prone to pitting corrosion, as shown in the ST0% sample in Figure 11. With the increase in the deformation amount, the boundary of the molten pool and grain boundary is destroyed, resulting in a large number of substructures, which leads to pitting corrosion mostly in other locations. The results show that the microstructure of SLM304 stainless steel has an important influence on its pitting corrosion behavior. Therefore, after deformation, the corrosion resistance of SLM304 stainless steel decreases due to the combined effect of many factors, such as interface damage, martensitic transformation, and destruction of the passive film.

To sum up, after the SLM304 stainless steel undergoes plastic deformation, significant changes occur in the microstructure, such as grain characteristics, phase composition, and cellular structure. Additionally, the characteristics of the passivation film on the surface of SLM stainless steel also slightly changes. The combined effect of the complex microstructure makes the nanohardness of SLM304 stainless steel increase with the increase in the deformation amount, while its corrosion resistance deteriorates. This study not only reveals the evolution mechanism of non-equilibrium metal microstructures and properties but also provides a theoretical reference value for the process innovation and engineering application of additive manufacturing technology.

## 5. Conclusions

This paper subjected SLM304 stainless steel to stretch deformation and investigated the microstructure, mechanical properties, and corrosion resistance of stainless steel with different deformation amounts. The following conclusions can be drawn:

(1) After stretch deformation of the SLM304 stainless steel, as the deformation amount increases, the grains are destroyed, leading to disordered grain orientation, and a large number of subgrain structures are generated. Moreover, the shape of the micron cellular structure changed and the size decreased.
(2) With the increase in the deformation amount, the content of α’ martensite increased significantly, and the thickness of the passive film on the surface of SLM304 stainless steel decreases slightly.
(3) With the increasing deformation amount, the nanohardness of SLM304 stainless steel gradually increases, and its corrosion resistance gradually deteriorates.
(4) The destruction of grains, martensitic transformation, dislocation accumulation, cellular structure, and changes in surface passive film characteristics caused by deformation lead to the increase in nanohardness of SLM304 stainless steel but cause the deterioration of its corrosion resistance.

## Figures and Tables

**Table 1:** SLM304 stainless steel composition (wt.%).

Material	Si	Mn	P	S	Cr	Ni	Fe
SLM304	0.36	1.12	0.02	0.02	18.19	8.61	Bal.

**Table 2:** Tensile mechanical properties of printed SLM304 stainless steel.

Material	Yield Strength σ_s_/MPa	Tensile Strength σ_b_/MPa	Elongation δ/%
SLM304	490	740	51

**Table 3:** Dynamic polarization test results of SLM304 stainless steel with different stretch deformation amounts in 3.5% NaCl solution.

Samples	E_pit_ (mV_SCE_)	E_cos_ (mV_SCE_)	I_pit_ (μAcm^−2^)	I_cos_ (nAcm^−2^)
ST0%	127.17 ± 3.5	−251.34 ± 6.5	5.56 ± 0.6	0.51 ± 0.05
ST10%	67.35 ± 1.7	−281.56 ± 6.1	9.53 ± 0.9	1.49 ± 0.15
ST20%	19.11 ± 0.5	−289.08 ± 6.3	10.94 ± 1.1	3.01 ± 0.2
ST30%	−51.91 ± 1.5	−296.28 ± 6.7	23.88 ± 2.4	5.24 ± 0.5
ST40%	−150.28 ± 4.3	−310.43 ± 6.5	67.45 ± 6.5	28.84 ± 2.5

E_pit_: pitting potential; I_cos_: corrosion current density; E_cos_: corrosion potential; and I_pit_: pitting current density.

## Data Availability

All data that support the findings of this study are included within the article.
